# A search for genetic diversity among Italian Greyhounds from Continental Europe and the USA and the effect of inbreeding on susceptibility to autoimmune disease

**DOI:** 10.1186/s40575-015-0030-9

**Published:** 2015-10-30

**Authors:** Niels C. Pedersen, Hongwei Liu, Angela Leonard, Layle Griffioen

**Affiliations:** Center for Companion Animal Health, School of Veterinary Medicine, University of California, One Shields Avenue, Davis, CA 95616 USA; 10358 Onondaga Rd, Onondaga, MI 49264 USA; 6687 S 1530 E, Salt lake city, UT 84121 USA

**Keywords:** Italian Greyhound, Genetic bottleneck, Inbreeding, Autoimmune disease

## Abstract

**Background:**

Previous studies documented the problem of inbreeding among Italian Greyhounds (IG) from the USA and its possible role in a multiple autoimmune disease syndrome. The present study is an extension of these earlier experiments and had two objectives: 1) to identify pockets of additional genetic diversity that might still exist among IG from the USA and Continental Europe, and 2) to determine how loss of genetic diversity within the genome and in the dog leukocyte antigen (DLA) complex relates to the problem of autoimmune disease in IG from the USA. Genetic testing was conducted using 33 short tandem repeat (STR) loci across 25 chromosomes and 7 STR loci that associated with specific dog leukocyte antigen (DLA) class I and II haplotypes. Standard genetic assessment tests based on allele frequencies and internal relatedness (IR) were used as measures of breed-wide and individual heterozygosity.

**Results:**

The results of these tests demonstrated that IG from the USA and Continental Europe belonged to a single breed but were genetically distinguishable by genomic allele frequencies, DLA class I and II haplotypes, and principal coordinate analysis (PCoA). In the second part of the study, 85 IG from the USA that had suffered various autoimmune disorders (case) and 104 healthy dogs (control) of comparable age were studied for genetic associations with disease. Case dogs were found to be significantly more homozygous in the DLA regions than control dogs. Principal coordinate analysis did not differentiate case from control populations. No specific STR-associated DLA-class I or II haplotype was associated with increased autoimmune disease risks. Reasons for the loss of genetic diversity and increased homozygosity among IG from the USA were studied using registration data and deep pedigrees. The breed in the USA started from a small number of founders from Europe and has remained relatively isolated and small in numbers, limiting breeding choices especially in the period before modern transportation and artificial insemination. An additional cause of lost diversity and increased homozygosity has been the influence of famous sires and their show-winning progeny. The most influential of these sires was Ch. Dasa’s King of the Mountain (King) born in 1978. Virtually all contemporary IG from the USA have King at least once in 10 generation pedigrees and 18 % of the genome of contemporary IG from the USA is shared with King.

**Conclusions:**

It was concluded that artificial genetic bottlenecks have concentrated numerous genetic polymorphisms responsible for autoimmune disease and that these risk factors did not originate in a specific individual or bloodline of the breed. Rather, they were of ancestral origin in both purebred and random bred dogs and inherited by descent. Italian Greyhound breeders in the USA have several options to improve breed health: 1) breed against homozygosity within the genome and in the DLA region, 2) avoid breeding dogs that have suffered an autoimmune disorder, 3) increase diversity by incorporating the genetic differences that exist in IG from Continental Europe, or 4) outcross to other small sighthound breeds. The latter two approaches must be undertaken with care to avoid introduction of new deleterious traits and to maximize retention and dissemination of new genetic diversity.

## Lay summary

Italian Greyhounds (IG) in the USA began with a small number of founders imported from Europe, with limited introduction of new dogs in the subsequent decades resulting in narrow breeding choices. These genetic bottlenecks were enhanced by a series of popular sires and their progeny, each followed by an extended period of close linebreeding. The resultant inbreeding has reduced genetic diversity, and increased homozygosity, in contemporary dogs. This has resulted in a greater susceptibility to a wide range of autoimmune diseases and inadvertent positive selection of other complex and simple deleterious traits. This loss of genetic diversity prompted a search for dogs currently in Continental Europe, parts of which were once behind the Iron Curtain. However, Italian Greyhounds from Continental Europe also lacked genetic diversity, but were genetically distinguishable from North American IG, both across the genome, and also in the DLA region. The cause of their lost diversity was not determined, although they probably suffered some of the same types of genetic bottlenecks as IG in the USA. Italian Greyhounds from Continental Europe appear to suffer from many of the same common heritable health problems as IG from the USA, but no information was obtained pertaining to the incidence of autoimmune diseases. The incidence of autoimmune diseases in IG from the USA may decrease with a concerted effort to decrease homozygosity across the genome and especially in the DLA region. Dogs that have suffered autoimmune disorders should not be used for breeding. Italian Greyhounds from the USA and Continental Europe, together with other small sight-hound breeds, could also be used to increase genetic diversity in both populations. However, care should be taken to avoid introductions of polygenic or simple recessive genetic disorders that may be unique to one population or the other.

## Background

A wide range of autoimmune disorders affect dogs, and in particular pure breeds [1]. Some autoimmune disorders are limited to a certain breed or breeds, while others occur in many different pure as well as mixed-breeds [2]. Necrotizing meningoencephalitis is a serious problem in Pug dogs [3]; pancreatic acinar atrophy [4], anal furunculosis [5], and chronic superficial keratitis [6] are relatively unique to German Shepherd Dogs, while sebaceous adenitis and Addison’s disease are common in a number of other breeds [7–9]. Italian Greyhounds from the USA are particularly prone to autoimmune disease and manifest most of the spectrum of autoimmune disorders recognized in dogs and humans [10]. Thyroiditis, which is the major cause of hypothyroidism, is one of the most common and widespread of autoimmune disorders among both purebred and random bred dogs [2].

Although it is generally accepted that autoimmune disease in both dogs [11–13] and humans [14, 15] involve the interactions of numerous genes and gene pathways, it is difficult to identify significant and universal disease-causing genotypes. As stated by Goris and Liston for humans [14] – “……. the genetic architecture of multifactorial autoimmune diseases is highly complex. Susceptibility rather than causality results from the combined effects of many variants, most of them common in the general population, that each exert a small effect on risk. Many different combinations of risk alleles are able to independently generate a high level of disease risk, without individual loci being necessary or sufficient for the development of disease.” The risk for autoimmune disease is higher in close relatives of affected individuals, because they inherit a greater number of these risk alleles [16]. However, only about one half of the risk is heritable, even between dizygotic or monozygotic twins, with the remainder being attributed to epigenetic and environmental factors [15, 16].

Although multiple genetic associations in various regions of the genome have been linked to autoimmune disease in humans, the strongest single genetic contributor of risk for autoimmune disease in people involves the human leukocyte antigen (HLA) complex [14, 15]. The HLA and DLA regions are gene dense and 40 % or more of the genes in this region of humans, and presumably dogs, are involved with immune functions [14]. It is not surprising; therefore, that many autoimmune diseases of dogs have also been associated with certain DLA class I or II [3, 4, 6, 11] haplotypes that confer significantly increased risk. The strongest DLA class I risk (OR = 17) was demonstrated for pancreatic acinar atrophy in German Shepherd Dogs [4], while the strongest DLA class II risk (OR = 12.75) was shown for NME in Pug Dogs [3].

It is evident from human studies, that risk for autoimmune disease also involves regions outside of the major histocompatibility complex [14, 15]. Genetic polymorphisms causing partial T-cell immunodeficiency, abnormalities in the function of regulatory T cells, and variations in cytokine signaling have all been implicated in autoimmune disorders [14]. If a large number of potential autoimmune disease associated polymorphisms exist in normal dogs, as suggested for humans [14], what might be a mechanism to bring these various risk factors together? The risk for autoimmune disease is higher in pure breeds of dogs and the incidence of disease increases as a breed loses genetic diversity. Therefore, a common thread in autoimmune disease of dogs is inbreeding. The present study focused on the relationship between the loss of genetic diversity and increased homozygosity both within the genome and in the class I and II regions of the DLA using STR markers and a spectrum of autoimmune disorders in IG. Extensive and multi-generational pedigrees and registration data were also used to trace for potential genetic bottlenecks that resulted in inbreeding.

## Results

### I. Genetic characterization of Italian Greyhound populations from the USA and Continental Europe

#### Genetic diversity statistics based on 33 genomic STR markers

**Fig. 1:**
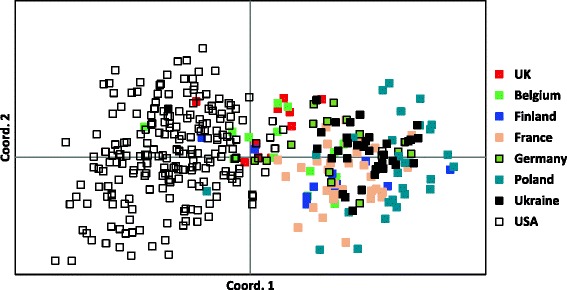
PCoA plot showing population structure of IG from the USA and from several countries in Continental Europe and the UK

Allele frequencies for 33 genomic STR markers of 213 IG from the USA and 174 from Continental Europe are listed in Table 1. Italian Greyhounds from the USA had a similar average number of alleles (Aa) at each of the STR loci (6.45 vs 6.26) compared with dogs from Continental Europe and a somewhat higher number of average effective alleles (Ae) per locus (3.37 vs 3.09) (Table 2). The European dogs were somewhat more heterogeneous than USA dogs (Ho = 0.624 vs 0.607). The positive fixation index or inbreeding coefficient (FIS) value for IG from both the USA and Europe indicated a degree of population substructure caused by individuals that were more inbred than their populations as a whole, and more so for American than Continental European IG. A PCoA plot was used to further differentiate USA and Continental European dogs (Fig. 1). Although IG from all of these regions clearly belonged to a single breed, dogs from the two disparate geographic areas demonstrated considerable population differentiation, with dogs from the USA clearly distinguishable from those of Continental Europe. Only one IG clearly of Continental European ancestry was found among IG from the USA and only seven IG from Belgium, UK, Ukraine, Finland and Poland were clearly of American ancestry. Dogs from Belgium, Germany, France and the Ukraine were similar to each other; IG from the UK and a portion of dogs from Belgium appeared to be a mixture of American and European dogs, while Polish IG tended to form a distinct subpopulation (Fig. 1).

**Table 1:** Nomenclature for 33 genomic STR loci, alleles and their frequencies in Italian Greyhound from Continetal Europe (*n* = 174) and the USA (*n* = 213). Frequencies for IG are listed in order: Continental Europe/USA

FH2054	AHTh171-A	FH2001	REN169D01	AHTH130	INU005	AHT137
152(0/.002)	219(.02/.01)	132(.3/.1)	202(.1/.11)	119(.41/.29)	106(.02/0)	131(.03/.09)
156(.06/.01)	225(.02/.03)	136(.01/.01)	210(.06/.22)	121(.27/.2)	120(.01/.01)	133(.1/.09)
160(.12/.1)	227(.16/.37)	140(.003/0)	212(.04/.02)	127(.15/.24)	122(.01/0)	135(0/.01)
164(.07/.09)	233(0/.002)	144(.1/.06)	214(.003/.002)	129(.01/.15)	124(.47/.5)	137(.23/.07)
168(.42/.18)	235(.01/.01)	148(.58/.81)	216(.28/.43)	131(.06/.01)	126(.29/.43)	141(.09/.09)
172(.28/.34)	237(.79/.58)	152(.01/.03)	220(.52/.22)	137(.11/.11)	128(0/.002)	143(.43/.38)
176(.06/.24)				141(0/.002)	130(.19/.05)	147(.12/.21)
180(0/.03)					132(.01/.002)	151(.003/.08)
184(0/.01)						
INU055	REN169O18	FH2848	REN105L03	REN54P11	REN64E19	REN247M23
204(.09/.04)	162(.52/.13)	228(.04/.01)	227(.06/.06)	222(.06/.19)	139(.07/.01)	268(.27/.33)
210(.47/.24)	164(.2/.07)	232(.07/0)	229(.01/0)	226(0/.02)	143(.39/.51)	270(.33/.24)
214(.13/.55)	166(.06/.002)	236(.08/.03)	231(.32/.15)	228(.12/.02)	145(.24/.25)	272(.16/.15)
218(.31/.17)	168(.19/.41)	238(.2/.22)	233(.59/.5)	232(.46/.34)	147(.15/.24)	274(.14/.28)
222(0/.002)	170(.03/.38)	240(.41/.71)	239(.003/0)	234(.07/0)	149(.15/0)	276(.11/0)
		244(.21/.03)	241(.02/.3)	238(.29/.42)		
AHTk253	INRA21	INU030	C22.279	LEI004	REN162C04	AHTk211
286(.08/.23)	95(.52/.6)	144(.22/.14)	116(.11/.02)	95(.58/.69)	202(.29/.2)	87(.53/.64)
288(.66/.56)	97(.01/.19)	148(0/.01)	118(.003/.01)	107(.42/.3)	204(.02/.01)	89(.06/.01)
290(.09/.02)	99(0/.01)	150(.76/.85)	124(.89/.97)	113(0/.01)	206(.69/.79)	91(.14/.16)
292(.16/.19)	101(.47/.19)	152(.02/.002)	128(0/.003)			95(.27/.2)
AHT121	AHTh260	VGL0910	VGL1063	VGL1165	VGL2918	
96(.19/.14)	238(0/.01)	13(.21/.01)	8(.046/.16)	18(.07/0)	7(.026/.02)	
98(.08/.16)	240(.29/.36)	14(.05/.01)	11(0/.01)	19(.37/.27)	12(.08/.05)	
100(.010/.29)	242(.003/0)	15(.01/.04)	12(.01/.003)	20(.003/.06)	13(.42/.072)	
102(.18/.2)	244(.04/.04)	16(.003/.09)	13(.28/.26)	21(.01/.02)	14(.03/.2)	
104(.06/.03)	246(.29/.17)	17(.44/.4)	14(.29/.39)	23(.04/.16)	15(.11/.07)	
106(.31/.06)	248(.01/0)	18(.02/.07)	15(.03/.01)	24(.01/.12)	16(0/.003)	
108(.003/.07)	250(.28/.01)	19(.17/.27)	17(.003/0)	25(.1/.16)	17(.003/.02)	
110(.05/.01)	252(.003/.03)	20(.07/.09)	18(.19/.02)	26(.01/0)	18(.19/.22)	
112(.01/.05)	254(.08/.39)	21(.04/.02)	19(.16/.14)	29(.38/.13)	19(.13/.01)	
114(.01/0)	256(0/.002)		20(.01/.002)	30(.003/.09)	20(.01/.17)	
				31(0/.01)	21(.003/.16)	
					22(0/.01)	
					23(0/.01)	
VGL0760	VGL1828	VGL2009	VGL2409	VGL3008	VGL3235	
19(.01/.002)	14(0/.02)	9(.26/.06)	13(.03/.09)	15(.03/.22)	13(.003/0)	
20(.51/.07)	15(.03/.09)	10(.07/.27)	15(0/.04)	16(.22/.05)	14(.64/.2)	
21(.28/.64)	16(.15/.01)	11(.1/.19)	16(.003/.03)	17(.22/.26)	15(.08/.15)	
22(.04/.12)	17(.08/.11)	13(.39/.46)	17(.14/.12)	18(.38/.2)	16(0/.01)	
23(.15/.17)	18(.14/.34)	14(.18/.02)	18(.67/.35)	19(.15/.26)	17(.26/.37)	
24(.003/.01)	19(.42/.41)	15(.01/.002)	19(.16/.365)	20(.003/.01)	18(.01/.25)	
25(.003/0)	20(.07/.02)	16(0/.01)	20(0/.01)	21(0/.01)	19(.003/.02)	
	21(.12/.003)				20(0/.002)	
					21(0/.002)

**Table 2:** Genetic assessment of Italian Greyhound from USA and Continental Europe using 33 genomic STR markers

	N		Aa	Ae	Ho	He	FIS
USA	213	Mean	6.452	3.368	0.607	0.641	0.053
		SE	0.431	0.226	0.026	0.026	0.008
EU	174	Mean	6.262	3.089	0.624	0.638	0.020
		SE	0.386	0.150	0.021	0.021	0.010
Total	387	Mean	7.238	3.513	0.615	0.665	0.073
		SE	0.528	0.214	0.022	0.023	0.006

*N* = # dogs; Aa = average alleles/locus; Ae = average effective alleles/locus; Ho = observed heterozygosity; He = expected heterozygosity; FIS = inbreeding coefficient

#### Internal relatedness among Italian Greyhounds

**Fig. 2:**
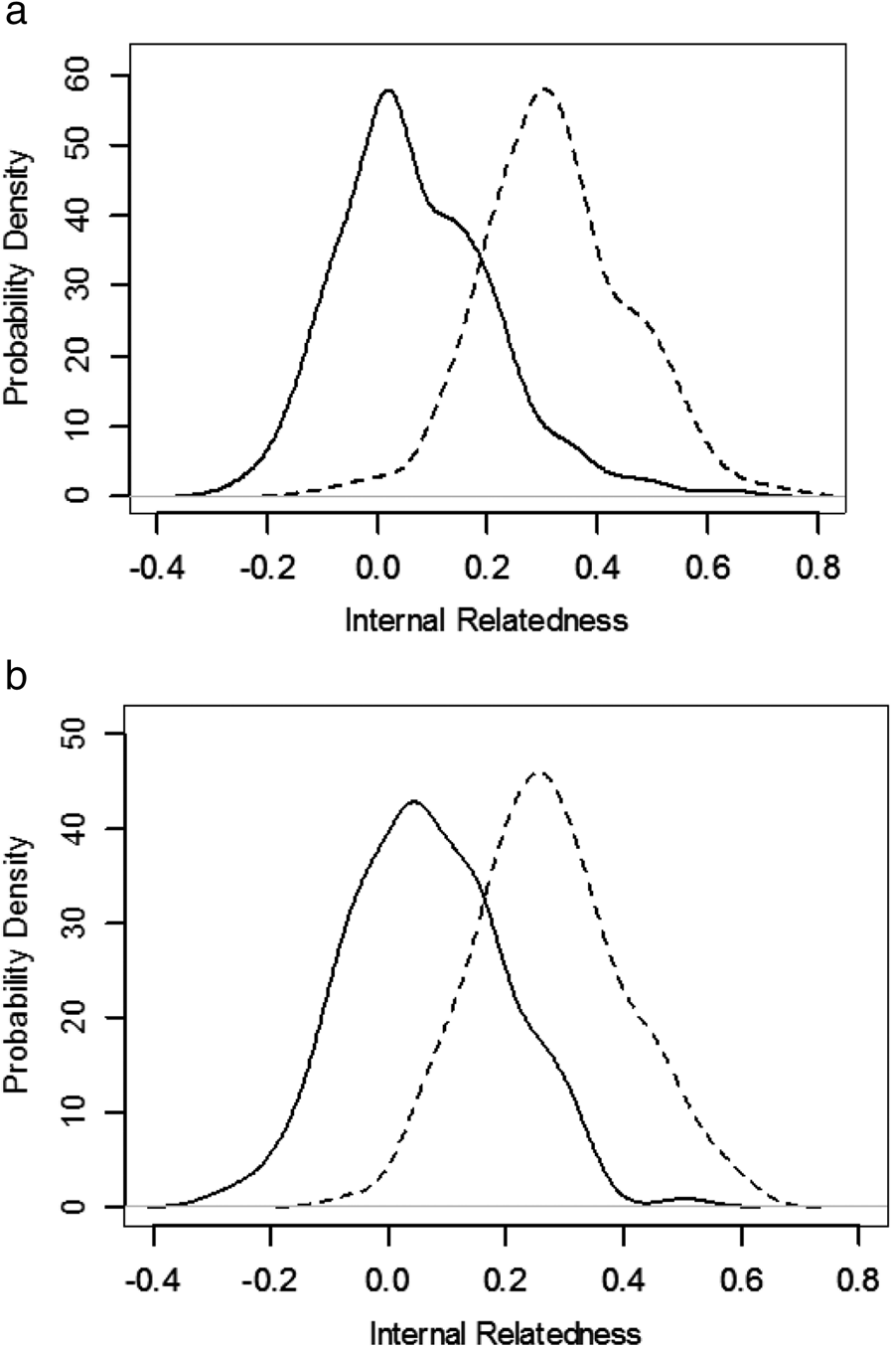
Distribution of IR estimates in IG from USA **a** and Continental Europe **b** based on intra-breed diversity (solid line), compared with IR adjusted for diversity lost during breed development (dashed line)

**Fig. 3:**
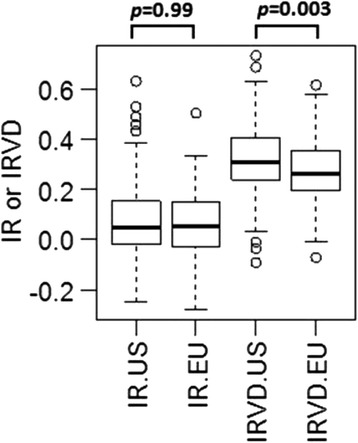
A comparison of IR or IRVD based on 33 genomic STRs between IG from USA (*n* = 213) and Europe (*n* = 174). Data were analyzed with one-way ANOVA and all possible pair-wise comparison was performed by TukeyHSD with 95 % confidence interval in R

Internal relatedness based on 33 genomic STRs for IG from the USA and Continental Europe is shown in Fig. 2. In this type of plot, a value of −1.0 would represent dogs from parents that were completely unrelated (shared no alleles) at all 33 genomic STR loci, while a value of 1.0 would indicate that the parents were genetically identical. The average expected IR value for offspring of full sibling parents is 0.25, assuming that the parents of the full siblings were randomly bred. The peak average IR values for IG from the USA were around 0.07, but the peak was biphasic with about one-half of dogs ranging from 0.07 to 0.20 and five individuals scoring >0.4. Some of these highly inbred individuals were seen as outliers on a box and whisker plot (Fig. 3). The peak average IR value for IG from Continental Europe was identical at 0.07, but the peak was less biphasic. Only one IG from Continental Europe had an IR values >0.40 compared to five IG from the USA (Fig. 3). However, the mean IR values were the same between populations (Fig. 3).

Internal relatedness values described above were based on alleles and allele frequencies measured in contemporary IG and did not reflect genetic diversity that has been lost during breed evolution. This lost diversity can be approximated by using allele frequency data from indigenous or village dogs that currently reside in the Middle East, SE Asia, and Pacific nations such as Taiwan, New Guinea, Bali, Australia, Brunei and the Philippines [18, 19]. Village dogs are the most genetically diverse population of dogs that has been studied by the investigators and the most likely to reflect the ancestral genetic diversity of modern breeds [18]. This approximation adjusts the frequency of an allele in IG to what would be found for that same allele in village dogs and was referred to as “IR village dog” or IRVD. The IRVD curve for IG from the USA had a peak value of nearly 0.32, while the peak IRVD for IG from Continental Europe was significantly lower at 0.28 (Fig. 3). The average IRVD values for offspring of full sibling village dogs would be 0.25, assuming that the parents were of random diversity. Very few IG from either the USA or Continental European had IRVD values below 0.

#### Genetic diversity statistics based on STR-associated DLA class I and II haplotypes

There were noticeable differences in the actual DLA class I and II haplotypes and their frequencies between IG from the USA and Continental Europe (Tables 3 and 4). Although Continental European IG possessed 14 DLA class I and 12 DLA class II haplotypes, and IG from the USA possessed similar numbers at 15 and 14, respectively, several haplotypes were unique to one population or the other (Tables 3 and 4). Continental European IG made effective use of fewer DLA class I and II haplotypes, with 1040, 1044 and 1052 comprising about 75 % of class I haplotypes, while 1008, 1044, 1052 and 1053 made up 72 % of the class I haplotypes in IG from the USA (Table 3). The dominant DLA class II haplotypes in Continental European dogs were 2017, 2034 and 2039 (78 %), while 2017, 2029, 2032, 2034, 2035 and 2036 (83 %) were dominant among IG from the USA (Table 4). STR-associated DLA class II haplotypes correspond to published haplotypes as determined by exon 2 sequencing of *DLA-DRB1, DQA-1* and *DQB-1* as shown in Table 4. Simple mutations in some STR loci led to situations where two different STR-associated DLA class II haplotypes were associated with the same sequence based haplotype (Table 4). The sharing of two major DLA class I and II haplotypes reflects a common heritage of the two populations. Linkage between class I and II haplotypes was strong, forming 20 extended haplotypes across the populations. Homozygosity in class I haplotypes was mirrored by homozygosity in the linked class II haplotypes. Specific linkages tended to reflect the frequency of class I or II haplotypes in the population, although some recombination was evident, e.g., haplotypes 1044/2034, 1052/2017, 1059/2035, 1059/2029 and 1053/2036 occurred in about 70 % of IG from the USA, with 1059 being linked to either 2029 or 2035.

**Table 3:** STR-associated DLA class I haplotype frequencies among Italian Greyhound from Continental Europe (*n* = 174 dogs and 348 haplotypes) and the USA (*n* = 213 dogs and 426 haplotypes)

VGL #	Class I Haplotype	EU	US
1008	386/373/289/182	0.03	0.21
1012	388/369/289/188	0.01	0.01
1016	382/371/277/178	0.10	0.06
1030	380/373/293/178	0.01	0.03
1036^b^	389/365/289/180	0.003	0.00
1040	380/371/277/186	0.24	0.04
1044	375/373/291/178	0.32	0.21
1048^a^	380/370/289/184	0.00	0.02
1049^a^	380/370/289/186	0.00	0.002
1050^a^	380/371/289/182	0.00	0.002
1051^b^	380/371/289/184	0.01	0.00
1052	380/372/289/184	0.20	0.19
1053	382/377/277/186	0.03	0.11
1054^a^	382/379/277/184	0.00	0.01
1055^a^	386/373/289/180	0.00	0.002
1056	386/373/289/190	0.01	0.01
1058^b^	387/378/287/186	0.01	0.00
1059	390/371/291/182	0.03	0.09
1065^b^	380/371/277/181	0.003	0.00

^a^present only in IG from USA

^b^present only in IG from Continental Europe

**Table 4:** STR-associated DLA class II haplotype frequencies among Italian Greyhound from Continental Europe (*n* = 174 dogs and 348 haplotypes) and the USA (*n* = 213 dogs and 426 haplotypes). Recognized DRB1/DQA1/DQB1 haplotypes based on exon 2 sequencing that correspond with STR-associated haplotypes are listed when known

VGL #	Haplotype	EU	US	Associated DRB1/DQA1/DQB1 haplotypes
2003	343/324/282	0.01	0.01	01503/00601/02301
2015^c^	339/327/280	0.00	0.002	
2017	343/322/280	0.23	0.22	01101/00201/01303
2023	341/323/282	0.01	0.03	00601/005011/00701
2029	337/324/268	0.03	0.09	02901/00301/00401
2030^c^	339/322/268	0.00	0.002	
2031	339/322/282	0.10	0.06	01301/00101/00201 or dqb002v
2032^c^	339/323/280^a^	0.00	0.09	00101/00101/00201
2033^c^	339/323/282^a^	0.00	0.01	00101/00101/00201
2034	341/322/280	0.32	0.21	00603/00101/00802
2035	341/323/280	0.03	0.13	00601/005011/00701
2036^c^	341/327/276^a^	0.00	0.09	00203/00901/00101
2037^c^	341/327/280^a^	0.00	0.01	00203/00901/00101
2038^b^	345/324/280	0.03	0.00	
2039	345/327/276	0.24	0.05	00201/00901/00101
2040^b^	345/327/280	0.06	0.00	
2041^b^	349/321/280	0.003	0.00	
2044^b^	343/324/268	0.003	0.00	

^a^Different STR haplotypes linked to the same sequence based haplotype

^b^present only in IG from Continental Europe

^c^present only in IG from USA

### II. Genetic comparisons of healthy (control) and autoimmune disease afflicted (case) IG from the USA

#### Gender association

The control population contained 104 dogs with known gender, 59 female and 45 males (F:M = 1.31). The F:M ratio for case dogs varied greatly depending on the form of autoimmune disease that they manifested (Table 5). Females were 2–5 times more likely to develop an autoimmune disorder, with the exception of immune mediated polyarthritis (IMPA), which had a F:M ratio nearly the same as the control population.

**Table 5:** Incidence of disorders in 91 autoimmune Italian Greyhound from USA

Disease	Incidence^a^	Female	Male	F/M Ratio
Addison	9	6	3	2
IMHA	30	25	5	5
IMTP	12	9	3	3
IMPA	23	12	11	1.1
Meningitis	6	4	2	2
Pemphigus	10	7	3	2.3
Thyroiditis	11	8	3	2.7
Orchitis	2	NA	2	NA
Total affected^a^	103	60	31	1.9
Total healthy	104	59	45	1.3

^a^Twelve dogs suffered two different manifestations at different times and were therefore counted twice. Six of the 91 IG did not contribute DNA for testing

#### Genetic diversity statistics based on 33 genomic STR markers

**Fig. 4:**
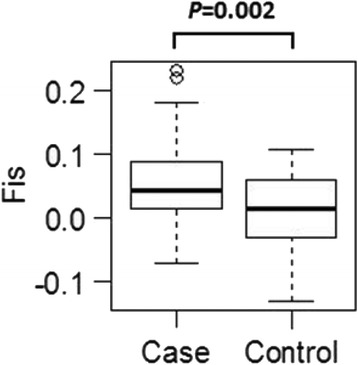
A comparison of FIS value based on 33 genomic STRs between the 85 autoimmune and 104 healthy control IG. Data were analyzed by paired *t*-test with 99 % confidence interval

**Fig. 5:**
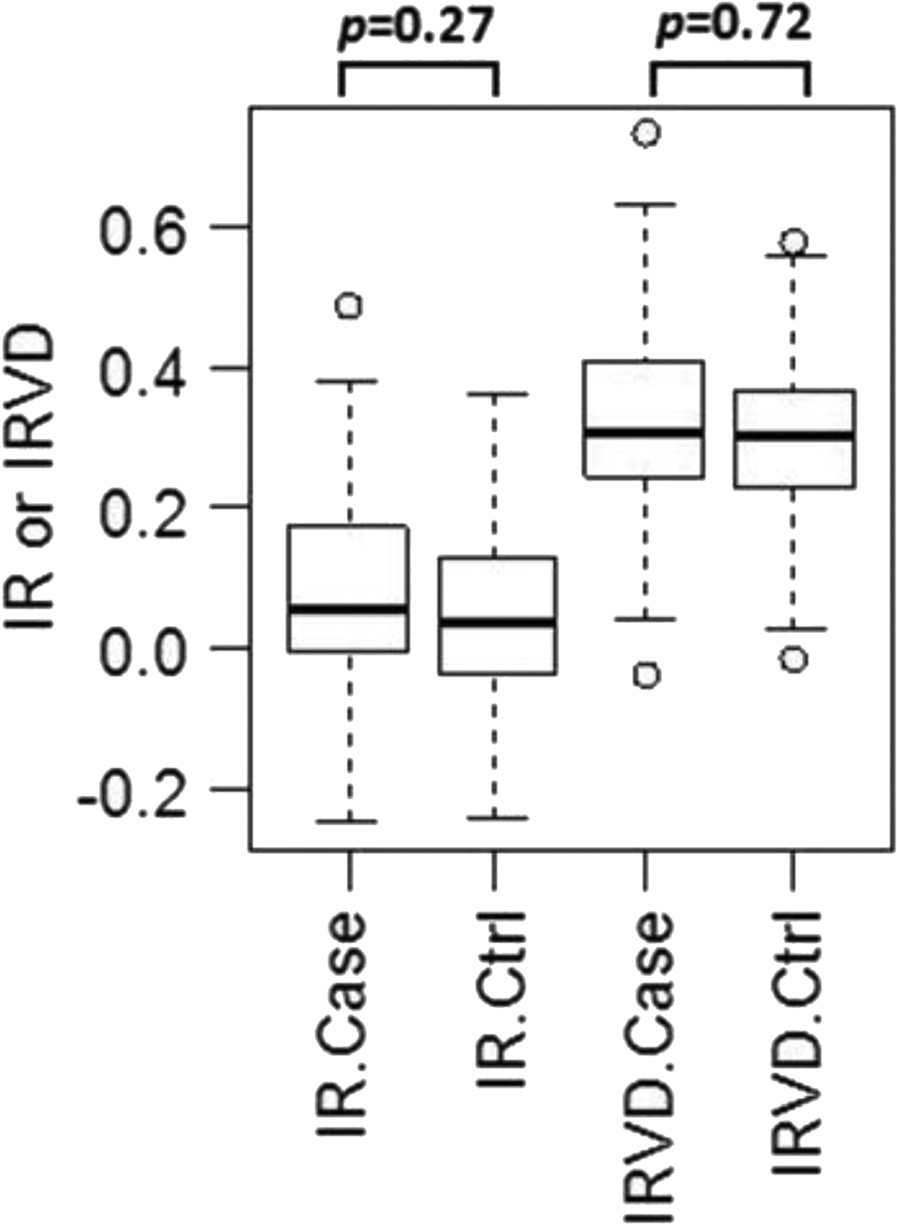
A comparison of IR or IRVD based on 33 genomic STRs between 85 autoimmune and 104 healthy IG from USA. Data were analyzed with one-way ANOVA and all possible pair-wise comparison was performed by TukeyHSD with 95 % confidence interval in R

The inbreeding coefficient (FIS) calculated from genomic STRs indicated that the control population was nearly in Hardy-Weinberg equilibrium, while the case population as a group was significantly more inbred than the healthy control dogs (Table 6; Fig. 4). However, there was no statistical difference in IR or adjusted IRVD scores between case and control dogs (Fig. 5).

**Table 6:** Genetic assessment parameters for healthy (control) and autoimmune disease affected (case) IG from USA

		N	Aa	Ae	Ho	He	FIS
Case	Mean	85	6.030	3.199	0.590	0.621	0.059
	SE		0.393	0.237	0.033	0.033	0.013
Control	Mean	104	5.455	3.099	0.609	0.613	0.009
	SE		0.372	0.229	0.032	0.030	0.010
Total	Mean	189	6.333	3.359	0.610	0.641	0.049
	SE		0.391	0.224	0.026	0.026	0.008

#### Principal coordinate analysis (PCoA)

**Fig. 6:**
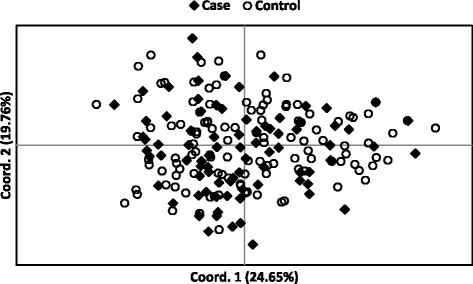
PCA based on 33 STR markers of 104 healthy control IG and 85 IG that suffered an autoimmune disease

The distribution of dogs with autoimmune disease and healthy controls in the entire population was also examined by PCoA (Fig. 6). Italian Greyhounds suffering from autoimmune disease could not be differentiated from the control population, supporting the previous IR comparisons.

#### DLA class I/II haplotype associations with autoimmune disease

The frequency of DLA class I and II haplotypes based on STRs in healthy and autoimmune disease affected IG from the USA was compared (Table 7). There was no specific STR-linked DLA class I or II haplotype that was significantly associated with risk for autoimmune disease. However, a moderate increase in relative risk (RR = 1.32–1.62, *p* <0.05.) was associated with homozygosity for alleles at 3/4 class I and 2/3 STR-associated DLA class II loci (Table 8). Significant and somewhat higher risk (RR = 1.83–2.54) was also observed for homozygosity at DLA class I and II haplotypes, respectively (Table 8).

**Table 7:** Frequency of DLA Class I & II haplotypes in 85 cases of autoimmune disease and 104 controls

	Class I			Class II	
Haplotype	Case	Control	Haplotype	Case	Control
1008	34(0.21)	48(0.23)	2003	1(0.01)	3(0.01)
1012	1(0.01)	3(0.01)	2017	33(0.20)	53(0.26)
1016	6(0.04)	13(0.06)	2023	5(0.03)	6(0.029)
1030	5(0.03)	6(0.03)	2029	19(0.12)	15(0.07)
1040	7(0.04)	7(0.03)	2030	0(0.00)	1(0.01)
1044	35(0.21)	39(0.19)	2031	6(0.04)	12(0.06)
1048	5(0.03)	4(0.019)	2032	14(0.09)	19(0.09)
1049	1(0.01)	0(0.00)	2033	3(0.018)	2(0.01)
1052	26(0.16)	47(0.23)	2034	35(0.21)	39(0.19)
1053	21(0.13)	22(0.11)	2035	20(0.12)	29(0.14)
1054	3(0.02)	2(0.010)	2036	15(0.09)	21(0.10)
1056	1(0.01)	2(0.010)	2037	3(0.02)	1(0.01)
1059	19(0.12)	15(0.07)	2039	10(0.06)	7(0.03)

**Table 8:** The frequency of homozygous alleles at individual DLA class I and II STR loci and at their associated haplotypes and risk for autoimmune disease in IG.

			Class I				Class II		Haplotype	
	#	3CCA	4ACA	4BCT	1131	5ACA	5ACT	5BCA	DLA-I	DLA-II
Case	85	33(0.39)	41(0.48)	40(0.47)	37(0.44)	41(0.50)	38(0.46)	51(0.61)	26(0.32)	24(0.29)
Control	104	23(0.22)	32(0.31)	37(0.36)	28(0.27)	35(0.34)	34(0.33)	44(0.42)	18(0.17)	12(0.12)
RR		1.76	1.57	1.32	1.62	1.49	1.42	1.470	1.83	2.54
*p* value		**0.014**	**0.015**	0.11	**0.018**	**0.025**	0.058	**0.007**	**0.024**	**0.004**

Bolded *p* values were <0.05

**Fig. 7:**
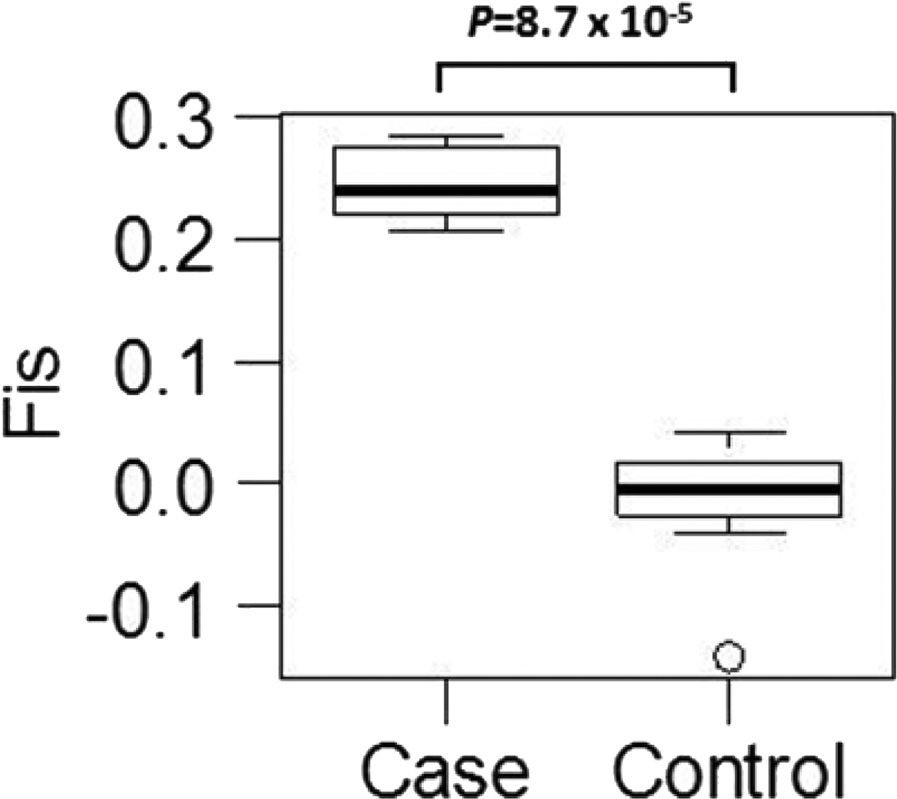
A comparison of FIS value based on 7 DLA class I and II STR loci between the 85 autoimmune and 104 healthy IG. Data were analyzed by paired *t*-test with 99 % confidence interval

Genetic assessment based on alleles at the seven DLA class I and II associated STR loci showed FIS values to be significantly higher for case dogs with autoimmune disease than for healthy control dogs (Fig. 7). FIS values for DLA-associated STR loci were higher in case dogs than for genomic markers (0.212 vs. 0.059) (Table 6, Fig. 7), indicating that homozygosity in the DLA class I and II was an even greater risk factor than homozygosity in other parts of the genome.

### III. Pedigree analyses Italian Greyhounds from the USA

#### Origin of Italian Greyhounds in the USA

The first IG in the USA were registered in 1886 and the number registered increased slowly until 1949, after which the breed enjoyed a steady increase in popularity (Table 9). Although only a small number of dogs were initially imported from Europe, further introgression of Continental European dogs was apparently limited as American breeders pursued a somewhat different appearance and temperament. The common origin, as well as the effect of subsequent population isolation, was confirmed by analyzing DNA of IG from Continental Europe and the USA. This was reflected in a PCoA plot of the two populations (Fig. 1) and in STR-associated DLA class I and II haplotype sharing as well as differences in allele usage (Tables 3 and 4).

**Table 9:** Registration statistics for IG in the USA 1886–2004

Years	#dogs	Years	#dogs	Years	#dogs	Years	#dogs
1886–91	41	1916–21	43	1946–51	237	1976–81	3215
1892–97	80	1922–27	46	1952–57	722	1982–87	4139
1898–03	29	1928–33	21	1958–63	2208	1988–93	8842
1904–09	51	1934–39	27	1964–69	3889	1994–99	10412
1910–15	41	1940–45	51	1970–75	3882	2000–04	13392

#### Popular sire effects

The IG breed in the USA started with a relatively few founders from Europe, creating the first artificial genetic bottleneck (Table 9). A severe artificial genetic bottleneck began in the 1980s, with the birth of King in 1978. In order to gauge his genetic contribution to contemporary IG in the USA, King’s influence over 10 generations was traced among dogs listed in the AKC Stud Book during each decade from 1970 to 2010 (Table 10). Three percent of 10 generation pedigrees in 1980s had King as an ancestor and this figure increased to 99.5 % by the 2000s.

**Table 10:** The percentage of pedigrees registered with the AKC and AKC Stud Book that listed Dasa’s King of the Mountain one or more times in a 10 generation pedigree in the decades 1970–2010.

	1970s	1980s	1990s	2000s	2010s
# Listed In AKC Stud Book	281	228	440	841	303
# IG King in 10 generations	0	7	384	837	302
% breeding dogs with King	0	3 %	87 %	99.5 %	99.7 %

Pedigrees were also analyzed for possible associations with King and autoimmune disease. Coefficients of inbreeding (COI) calculated from pedigrees mirrored what was found with DNA markers, i.e., case dogs had higher COI than control dogs, but significance was at the 93 % probability level (Table 11). The genetic contributions of *King* to case dogs trended higher than in control dogs, but only the mean King blood in a homozygous state approached significance at 90 % probability (Table 11).

**Table 11:** Genetic contributions of *King* based on 10 generation pedigrees and association with autoimmune disorders. Control dogs = 104; case dogs = 85

	COI(mean)	%King (mean)	%King Homozygosity (mean)
Case	15.38	18.83	2.10
Control	13.43	17.28	1.66
*p*-value*	0.071	0.275	0.1

## Discussion

The search for genetic diversity among IG concentrated on the USA and Continental Europe, because IG from these two regions have been long separated by geography, two World Wars and the Iron Curtain. Although there have been imports of European dogs to the USA over the decades, they have been few in numbers and their impact relatively small. A report in a breed publication listed 177 imports from other countries to the USA between 1945 and 1977, but 47 were from Canada and 66 from the UK [17]. The remaining 64 dogs were from Continental Europe and of non-American stock. Among all 177 imports to the USA, only contributions from Austria (11 dogs) led to champions in the USA and only a small number were ever registered as breeders. A small number of dogs were exported from Europe to the USA after 1977, but the small number of show winners and stud book registrations resulting from these imports suggests that their influence on the breed in the USA has been relatively small. Therefore, it was not surprising to find that IG from these two parts of the world were related as a breed, but genetically distinguishable by various parameters related to genomic and DLA associated marker.

A second and major objective of the study was to compare and contrast health problems between the two populations, and in particular to better understand genetic polymorphisms that might explain the high incidence of autoimmune disease that was documented earlier for IG from the USA [10]. Unfortunately, information pertaining to the incidence of autoimmune disorders among European IG was sparse and it was impossible to say whether it was a lesser problem or just not recognized. However, IG from Continental Europe appear to suffer from several other heritable disorders of the breed, including juvenile long bone fractures, congenital megesophagus, Legg–Calvé–Perthes Disease, color dilution alopecia, epilepsy, and possibly enamel hypoplasia and certain eye and heart problems (personal communications). Given a lack of knowledge on disease problems in IG from Continental Europe, genetic association studies for autoimmune disease were conducted using case and control dogs from only the USA.

The association between inbreeding and autoimmune disease in IG from the USA involved genetic comparisons between 85 affected (case) and 104 healthy (control) dogs. Although these populations were small compared to human studies of autoimmune disease, previous reports suggest that due to the unique makeup of their genomes, ~100 case and ~100 control dogs from a pure breed of dogs would be sufficient to detect genetic associations even in complex genetic traits such as those involved in autoimmunity [19]. The number of alleles per genomic STR loci and effective alleles per locus were not significantly different between case and control dogs, although FIS values were significantly higher for case than control dogs. This finding was identical to that observed in an earlier study on IG and autoimmune disease [10]. Principal coordinate analysis showed case and control populations to be congruent, which was different from Standard Poodles, where genetic outliers were largely free of autoimmune disease [20]. Unlike standard tests based on allele frequency, which are good at assessing populations, IR has been used to assess the relatedness of an individual’s parents [21] and to study the potential effect of inbreeding on disease and fitness [22–26]. Unadjusted IR and adjusted IRVD values of individual IG were also the same among healthy and autoimmune affected individuals. These results suggest that most IG in the USA possess a large number of risk factors for autoimmune disease and that any random selection of parents can produce one or more puppies with a higher than average susceptibility for autoimmune disease.

In spite of published relationship of certain DLA class I and II haplotypes with specific autoimmune diseases [3–6, 11, 27], we were unable to identify a specific STR-associated DLA class I or II haplotype that was significantly associated with autoimmune diseases in IG from the USA. There is evidence in dogs, as in humans [14, 15], that genetic risk associations exist both outside and within the DLA. A search for genetic associations with immune brain and spinal cord disease between Pug dog, Chihuahua and Maltese implicated both breed specific and shared risk polymorphisms in the DLA class II region, and on chromosomes 4 and 15 [12]. Eleven genes on five different chromosomes were found to be associated with SLE-like disease in dogs characterized by different types of antinuclear antibodies or steroid responsive meningitis [13]. Several genetic polymorphisms outside the DLA have been associated with increased risk for SLE in Nova Scotia Duck Tolling Retrievers [27]. Forty two putative genes associated with Addison’s disease in humans were investigated in seven affected breeds of dogs using gene specific SNP arrays, and potential associations identified in five breeds [9]. Ten candidate genes were found singly in one breed, nine genes had multiple putative associations within a breed, and one gene had the same two putative allelic associations in two breeds.

The strongest association with autoimmunity in IG from the USA was homozygosity at both genomic and DLA loci. FIS values based on observed and expected heterozygosity in the 33 genomic loci were significantly greater among case than control dogs, but even more so for the DLA region markers. The DLA region is rich in genes controlling immune responses and is in extended linkage disequilibrium. Therefore, it is possible that additional genes in extended linkage to DLA class I and II markers were also involved with autoimmunity in IG, especially when in a homozygous state.

What is the cause of the inbreeding observed in IG from the USA? The breed started with the introduction of a small number of dogs from Europe and expanded from that initial population with limited introgressions from Europe. The breed in the USA has remained relatively small for most of its existence and is currently ranked 72^nd^ in popularity by the American Kennel Club. This has made it more difficult to locate the most diverse mates in earlier times. However, the rapid nature of modern transportation and shipment of frozen semen should ease the problem of mate selection. The problem is not only a limited pool of potential mates, but a limited pool of dogs with both a desired genetic backgrounds and sound health. Although the limited founder population, lack of significant introgression with dogs from outside of the country and small population size have been factors favoring inbreeding, it is difficult to deny the effects popular sires and their progeny have had on further narrowing the available pool of genetic diversity. There have been several noteworthy popular sire influences on the breed in the USA during the last 6 decades. Ch. Ulisse di Peltrengo of Winterlea came to the USA via Italy and Scotland as an Italian and English champion [28]. “Ulisse di Peltrengo of Winterlea proved to be a most potent and welcome size-reducer. He had an impeccable front, exceptionally deep brisket, and most of all-he gave to his puppies an exciting elegance and dazzling showmanship [28].” He became a United States Champion in 1958 and sired 10 champions out of 9 different bitches. Four of his champion sons sired a total of 40 champions. Five of his great-grandsons, in a direct male-to-male lineage, accounted for 24 champions, and one great-great-grandson sired 8 champions. The show-winning form of Ulisse and his progeny were the most likely explanation for the sharp increase in IG registrations that occurred after 1960. The next, and possibly greatest, sire among IG from the USA was King, born April 9, 1978. The subsequent impact of this sire on IG in the USA was best described in a publication called *Born to Win, Breed to Succeed* [29] - “Ebony Queen was in good company, for her favorite mate [and half-brother] King of the Mountain sired 78 champions for the breed’s siring record and his son Ch. Mira Hill N’Dale D’Dasa’s sired 46 champions. If ever a producing line was a testimony to the idea of family importance in breeding of outstanding animals, it is this exceptional line of Italian Greyhound. Their collective ability to harmonize with the pedigrees throughout the breed makes their contribution one still strongly appreciated decades later.” The rise of King and his progeny was also associated with a second rise in IG registrations that occurred in 1982–2004. Pedigree studies confirm the rapidity with which King blood was incorporated into the breed and virtually all contemporary IG from the USA have the King in their 10 generation pedigrees and share around 18 % of his genome. The last of the popular sires for the breed in the USA was Ch. Tekoneva’s Dario. He was not of King’s heritage and was actively bred in the 1990s, probably in an attempt to reduce the genetic contributions of King blood-lines. However, show winners from his line came mainly from pairings with bitches heavy in King’s genes. One example was Ch. Windriver Ruby Tuesday, a popular bitch that was bred seven times to Ch. Tekoneva’s Dario (http://www.pedigreedatabase.com/italian_greyhound/dog.html?id=1961170-windriver-ruby-tuesday&p=simplechart&, accessed October 26, 2015).

It is tempting to implicate certain dogs or bloodlines for introducing a novel deleterious mutation into a breed. This may be the case for breed specific recessive mutations such as those responsible for enamel hypoplasia, PRA and glaucoma (https://www.vgl.ucdavis.edu/services/italiangreyhound.php, accessed October 26, 2015). Other disorders are brought into the breed by deliberate or surreptitious outcrossing with other breeds, such as terriers from England [30]. However, the autoimmune disorders occurring in IG also occur in many different random- and pure-breeds [2], suggesting that the genetic basis for autoimmune disease is deeply ancestral in dogs and only came to the forefront when the responsible genes were acquired by descent and further concentrated by inadvertent positive selection. Even though King, and important sires before and after him, did not bring autoimmune disease and other heritable disorders into the breed, their commanding appearance in the show ring and ability to breed show winners has had an irrefutable effect on the genetic diversity of IG in the USA, which in turn concentrated and amplified genetic polymorphisms responsible for increased risk.

The conclusion of this study was that inbreeding is a significant risk factor for autoimmune disease and mirrors that of a recent study on sebaceous adenitis and Addison’s disease in Standard Poodles that used an identical format [20]. Standard Poodles evolved from performance dogs over the last 500 years or so, while IG have been recognized as companions and household pets starting over 2000 years ago with the ancient Greeks. Therefore, IG have been subjected to human directed genetic manipulations longer than Standard Poodles. Both breeds have similar total and effective alleles per locus. Standard Poodles have almost three times more DLA class I and II haplotypes, but only one or two haplotypes predominate, whereas DLA haplotype frequencies in IG are more diverse. No specific DLA haplotype is associated with autoimmune disease in IG, while certain minor haplotypes in Standard Poodles are associated with some degree of risk or protection. However, there were differences in how inbreeding occurred in IG and Standard Poodles. Standard Poodles have a large registry that started with a much greater amount of genetic diversity, which then underwent a significant artificial genetic bottleneck in the last half of the twentieth century as a result of certain highly desired blood lines. IG from both Continental Europe and the USA started with a limited genetic base. IG in the USA were further affected by a series of popular sire effects, while it is likely that IG from Continental Europe were more influenced by geographic separation and small population sizes created by two World Wars and the Iron Curtain. Highly desired lines of Standard Poodles originated in North America in the mid-twentieth century were exported across the world and replaced most indigenous lines, including those that had been closeted behind the old Iron Curtain. In contrast, a genetically diverse population of IG still exists in Continental Europe and the two populations have had much less transoceanic introgression. The exception may be IG from the UK and Belgium, which tend to be mixtures of USA and Continental European lines. A long period of close line-breeding caused a marked imbalance in genetic diversity in Standard Poodles with 30 % of the diversity retained in 70 % of the dogs, while case and control populations of IG in the USA were genetically indistinguishable. Homozygosity both within the genome and in the DLA is a significant risk factor for autoimmune disease in IG from the USA but not for Standard Poodles.

Individuals, whether dogs or humans, possess a large mixture of protective and risk alleles [31–33]. This is true for both simple and complex (polygenic) traits. Why do normal individuals possess both protective and risk alleles for autoimmunity? Immunity to infectious agents is one of the strongest selection pressures in human evolution and several autoimmune-associated genes show signs of positive selection, favoring either the protective or risk allele, depending on the case [31–33]. Therefore, the genetic polymorphisms that favor a strong immune response against infections appear to be the same polymorphisms that increase or decrease risk for autoimmune disease.

How do you know when the health of a breed is in trouble? One can argue that it is when undesirable ancestral traits, both complex and Mendelian, reach troublesome levels and when breed specific deleterious simple recessive traits began to appear. How do you respond when a breed becomes overly inbred and health is unfavorably affected? The best solution is to never reach this point, which requires starting a breed with the widest possible genetic base and then diligently breeding to maintain that diversity. Unfortunately, this does not usually occur; one study of 26 breeds registered with the Swedish Kennel Club concluded that 90 % of initial genetic diversity of a breed is rapidly lost [34]. It is obvious that the impulse to attain a specific breed type is great and that the fastest way to reach this goal is to breed champions to champions and to emphasize popular sires and their progeny. This philosophy was unfortunately fixed in breeder’s minds by publications such as “Born to Win, Breed to Succeed “[29]. The various solutions have been well described and will vary from breed to breed [35]. The situation with IG in the USA is difficult, because it is uncertain whether the breed has retained significant overall genetic diversity to counteract small founder numbers, several popular sire effects, and a number of complex and simple deleterious traits. Breeding against homozygosity, either in the genome or in the DLA, is an obvious tactic that can be immediately used and based on this study, should improve health. The temptation to eliminate complex and simple recessive mutations should be undertaken with care, because it may cause significant loss of diversity. Fortunately, breeding against homozygosity may also lower the incidence of these recessive disorders. Outcrossing is another tactic that could involve greater use of IG from Continental Europe or similar breeds. The breed was reportedly negatively impacted by crossbreeding to decrease size in the 16^th^ century and had to be restored to its more ancient form by breeders in the 17^th^ century (https://en.wikipedia.org/?title=Italian_Greyhound, accessed October 26, 1015). Indeed, there are also anecdotal and scientific findings that suggest crossbreeding has occurred to some extent over the last century or less. It has been rumored that other breeds such as Chihuahua, Toy Manchester terrier, Miniature Pinscher, Fox Terrier and Whippet were used at an earlier time to bring in certain coat colors, size, and other desired phenotypic traits. The introduction of terrier blood, especially from England, has been a source of much concern [30]. Indeed, congenital megesophagus identical in clinical form to that observed in IG in the USA and Europe has been well researched in both Miniature Schnauzer and Wire Haired Fox Terriers [36, 37]. Color dilution alopecia has also come into the breed with the introduction of the black coat color, also most likely from Terriers in England. It may not be a coincidence that Chihuahuas are the only dogs that share the same six maternal lineages found in American IG [10].

## Conclusions

Italian Greyhounds (IG) in the USA began with a small number of founders imported from Europe and only limited introgression of new dogs from Europe thereafter. This genetic bottleneck was enhanced by both a lack of numbers and a series of popular sires and their progeny, each followed by an extended period of close linebreeding. The resultant inbreeding has reduced genetic diversity and increased homozygosity in contemporary dogs from the USA and has resulted in a greater incidence of a number of polygenic and simple recessive deleterious traits. This loss of genetic diversity among IG from the USA and its effect on breed health prompted a search for additional genetic diversity, particularly among IG found in Continental Europe, parts of which were sheltered behind the Iron Curtain. Italian Greyhounds from Continental Europe were found to be genetically distinguishable from IG in the USA with evident population substructure even between various countries. Although IG from Europe were genetically distinguishable from American IG, they were also found to lack genetic diversity, although somewhat less so than dogs from the USA. Unfortunately, no information on autoimmune disease problems was provided by breeders from outside the USA, thus negating a comparative study of autoimmune disease in both populations. Loss of genetic diversity among IG from the USA has led to the emergence of a subpopulation of IG that was significantly more inbred than healthy control dogs. The genetics of autoimmune disease were found to be complex and not related to any specific DLA class I or II haplotype. The increased risk for autoimmune disease among this subpopulation could not be attributed to any particular popular sire and was presumed to be ancestral in many pure and random bred dogs and inadvertently enhanced by positive selection for desirable phenotypic traits. The incidence of autoimmune diseases in IG from the USA may decrease with a concerted effort to minimize homozygosity across the genome and especially in the DLA region. Dogs that have suffered autoimmune disorders or are closely related to affected individuals should not be used for breeding. Italian Greyhounds from the USA and Continental Europe and other small sight-hound breeds could also be used to increase genetic diversity between and among their respective populations. However, much more needs to be learned about existing health problems in IG, and in particular IG from Continental Europe. Such information is necessary to avoid introductions of polygenic or simple recessive genetic disorders that may be unique to one population or the other.

## Methods

### Sample collection

DNA from IG was submitted in the form of buccal swabs or EDTA whole blood. Owners were also asked to complete a form listing details on the dogs’ pedigree, signalment and health (http://www.vetmed.ucdavis.edu/ccah/research/Italian_greyhound_autoimmune_study.cfm), accessed October 26, 2015. DNA for genetic testing was available from 213 IG from the USA and 175 mainly from Continental Europe including France (*n* = 33), Germany (*n* = 16), Belgium (*n* = 14), Italy (*n* = 6), Slovenia (*n* = 7), Romania (*n* = 4), Ukraine (*n* = 35), Latvia (*n* = 4), Russia (*n* = 4), Finland (*n* = 11), Poland (*n* = 28), Great Britain (*n* = 10), Norway (*n* = 2), and Sample collection was conducted under UC Davis IACUC protocol #16643.

### Case and Control Populations from USA

Eighty-five of IG from the USA suffered one or more autoimmune disorders (Table 5). Six additional IG suffering from autoimmune disease had insufficient DNA for genetic testing but were included for analysis of disease forms, age and gender. One hundred four dogs with no known history of autoimmune disease within three generations were selected as controls. Among the 91 case dogs, 60 are female and 31 male (Table 5) ranging from 1.4 to 15.2 years of age (mean 7.5 years.). A number of these dogs were reported previously as part of an earlier study on autoimmune disease in IG [10]. The healthy control population consisted of 59 female and 45 males with an age range of 0.8–16.3 years of age (mean 8.1 year.).

### DNA extraction

DNA was extracted from a single cytology brush by heating at 95 °C in 400 μl 50 mM NaOH for 10 min and the pH neutralized with 140 μl 1 M Tris–HCl, pH 8.0 [38]. Blood samples (200 μl) were extracted using QIAGEN QIAamp®DNA blood mini and midi kits (QIAGEN Inc., Valencia CA, USA).

### Genomic diversity testing

Thirty three STR loci across the canine genome were multiplexed into two panels (Table 1). Amelogenin gene primers for gender determination were also included [39]. Primers, dye labels, repeat motif, allele size range and known alleles for this set of markers have been previously published [20]. A second panel consisted of two additional di-STRs, FH2001 and LEI004 and 10 of 15 tetra-STRs validated for forensic testing [40]. Genotyping was conducted by the Veterinary Genetics Laboratory, UC Davis, and data were analyzed using STRand software [41]. STRs were also used to determine genetic differences within regions of canine autosome 12 encoding DLA class I (*DLA88*) and DLA class II (*DLA-DQB1, −DQA1* and *–DRB1*). The STR designations, forward and reverse primers, alleles/locus, and allele size ranges for the four DLA class I and three class II STRs have been previously published [20]. Specific alleles of the DLA class I and II STR loci were found to be strongly linked, forming distinct haplotypes as determined by analysis with Phase [42]. Further Phase analysis identified strong linkages between DLA class I-II haplotypes that proved helpful in correcting errors made by independent Phase analyses of each region. Each STR-associated DLA class II haplotype was associated with a specific international designated sequence based haplotype, as determined by comparing STR and exon 2 sequence based on haplotypes from IG previously tested [10].

STRs rather than single nucleotide polymorphisms (SNPs) were chosen for this study because of their power to discern genetic differences between individual dogs, breeds, and randomly breeding village (indigenous) dog populations in a rapid, reproducible and cost-effective manner using low quality DNA. The power or STRs in discerning genetic relationships has been demonstrated for African village dogs, where 89 STR loci yielded identical results to 300 SNP loci in principal component analysis and STRUCTURE [43]. STR loci have also been used to interrogate MHC class I and II polymorphisms in the dog [44, 45] and cat [46]. Previous studies have confirmed a strong correlation between haplotypes derived from exon 2 sequencing of DLA class II genes and haplotypes derived from linked STR loci in IG [10] and Standard Poodles [7].

### Statistical analyses

Allele frequencies from 33 genomic STR loci were used to determine average alleles/locus (Aa), average effective alleles/locus (Ae), observed heterozygosity (Ho), expected heterozygosity (He), inbreeding coefficient FIS and PCoA using GenAIEX 6.5 [47, 48]. All possible pair-wise comparison was performed by TukeyHSD with 95 % confidence interval in R.

Internal relatedness (IR) was measured based on the number and frequency of alleles at a locus as described by Amos et al. [21] and based on an earlier calculation of Queller and Goodnight [49]. Internal relatedness is a measure of heterozygosity that places more weight on uncommon alleles. Therefore, it is an indirect measure of the genetic relationship of an individual’s parents. The average IR values for offspring of full-sibling matings was 0.25 as determined by testing of offspring of inadvertent full-sibling matings, rather than 0.5 as predicted by the published equation [21]. Therefore, an IR value of 0.25 was used to measure relatedness equivalent to what would be expected among offspring of full sibling pairs. IR values were graphed in two manners: 1) comparing individual IG with other dogs in the population, and 2) adjusting the frequencies of STR alleles in individual IG with the frequency of the same alleles in village (indigenous) dogs. The second comparison accounted for potential loss of diversity that occurred as a result of breed development and closure of the registry. Village dogs from the Middle East, SE Asia and Pacific region are the most outbred population of dogs studied and have been used as a gold-standard for estimating loss of genetic diversity among modern breeds [18, 50].

### Pedigree analyses

Pedigrees of 45,308 IG were obtained from a database maintained by one of the authors (AL). This includes pedigrees on every IG that was registered and bred with the American Kennel Club (AKC) from 1884 to present and the Kennel Club (UK) and Canadian Kennel club from the 1880s to 1980s. In addition to pedigrees from the AKC, the database incorporates The Irish Kennel Club, stud pages, and show catalogs. Websites from several countries such as Australia and Norway have large lists of pedigrees on their IG pages. Additional pedigrees were obtained from Germany, Russia and Italy. About 80 % of pedigrees after 1950 go back at least 10 generations and cover 70–75 % of the IG that are being bred worldwide. This database is available to any researcher upon request.

Coefficients of inbreeding (COI) were calculated using complete pedigrees and CompuPed v.4.0 professional software. This version is no longer available and has upgraded to Millennium Pro (PedFast Technologies, Frankfort, IL, USA). The program can also calculate the total number of times that a particular dog appears in a 10 generation pedigree, the genetic contribution of specific ancestors [51], and the genetic contribution of a significant ancestor that exists in a homozygous state.

## References

[1] Pedersen NC (1999). A review of immunologic diseases of the dog. Vet Immunol Immunopathol.

[2] Bellumori TP, Famula TR, Bannasch DL, Belanger JM, Oberbauer AM (2013). Prevalence of inherited disorders among mixed-breed and purebred dogs: 27,254 cases (1995–2010). J Am Vet Med Assoc.

[3] Greer KA, Wong AK, Liu H, Famula TR, Pedersen NC, Ruhe A, et al. Necrotizing meningoencephalitis of Pug dogs associates with dog leukocyte antigen class II and resembles acute variant forms of multiple sclerosis. Tissue Antigens. 2010;76(2):110–8. 10.1111/j.1399-0039.2010.01484.x 20403140

[4] Tsai KL, Starr-Moss AN, Venkataraman GM, Robinson C, Kennedy LJ, Steiner JM, et al. Alleles of the major histocompatibility complex play a role in the pathogenesis of pancreatic acinar atrophy in dogs. Immunogenetics. 2013;65(7):501–9. 10.1007/s00251-013-0704-y PMC3857963 23604463

[5] Massey J, Short AD, Catchpole B, House A, Day MJ, Lohi H, et al. Genetics of canine anal furunculosis in the German shepherd dog. Immunogenetics. 2014;66(5):311–24. 10.1007/s00251-014-0766-5 24626934

[6] Barrientos LS, Zapata G, Crespi JA, Posik DM, Díaz S, It V, et al. A study of the association between chronic superficial keratitis and polymorphisms in the upstream regulatory regions of DLA-DRB1, DLA-DQB1 and DLA-DQA1. Vet Immunol Immunopathol. 2013;156(3–4):205–10. 10.1016/j.vetimm.2013.10.009 24238945

[7] Pedersen NC, Liu H, McLaughlin B, Sacks BN (2012). Genetic characterization of healthy and sebaceous adenitis affected Standard Poodles from the United States and the United Kingdom. Tissue Antigens.

[8] Hernblad TE, Bergvall K, Egenvall A (2008). Sebaceous adenitis in Swedish dogs, a retrospective study of 104 cases. Acta Vet Scand.

[9] Short AD, Catchpole B, Boag AM, Kennedy LJ, Massey J, Rothwell S, et al. Putative candidate genes for canine hypoadrenocorticism (Addison’s disease) in multiple dog breeds. Vet Rec. 2014;175(17):430. 10.1136/vr.102160 25124887

[10] Pedersen NC, Liu H, Greenfield DL, Echols LG (2012). Multiple autoimmune diseases syndrome in Italian Greyhounds: preliminary studies of genome-wide diversity and possible associations within the dog leukocyte antigen (DLA) complex. Vet Immunol Immunopathol.

[11] Jokinen P. Identifying genetic risk factors in canine autoimmune disease. University of Helsinki, 2011, [https://helda.helsinki.fi/bitstream/handle/10138/24488/identify.pdf?sequence=1] accessed October 26, 2015.

[12] Schrauwen I, Barber RM, Schatzberg SJ, Siniard AL, Corneveaux JJ, Porter BF, et al. Identification of novel genetic risk loci in Maltese dogs with necrotizing meningoencephalitis and evidence of a shared genetic risk across toy dog breeds. PLoS One. 2014;9(11):e112755. doi:10.1371/journal.pone.0112755. 10.1371/journal.pone.0112755 PMC4231098 25393235

[13] Wilbe M, Kozyrev SV, Farias FH, Bremer HD, Hedlund A, Pielberg GR, et al. Multiple Changes of Gene Expression and Function Reveal Genomic and Phenotypic Complexity in SLE-like Disease. PLoS Genet. 2015;11(6):e1005248. doi:10.1371/journal.pgen.1005248. 10.1371/journal.pgen.1005248 PMC4461293 26057447

[14] Goris A, Liston A. The immunogenetic architecture of autoimmune disease. Cold Springs Harbor Perspectives in Biology, 2012; doi: 10.1101/cshperspect.a007260. 10.1101/cshperspect.a007260 PMC3282406 22383754

[15] Wang L, Wang FS, Gershwin ME. Human autoimmune diseases: a comprehensive update. J Intern Med. 2015;278(4):369–95. 10.1111/joim.12395 26212387

[16] Selmi C, Lu Q, Humble MC (2012). Heritability versus the role of the environment in autoimmunity. J Autoimmun.

[17] Fretwell F (2013). Imports to the US. Ital Greyhound Mag.

[18] Pedersen N, Liu H, Theilen G, Sacks B (2013). The effects of dog breed development on genetic diversity and the relative influences of performance and conformation breeding. J Anim Breed Genet.

[19] Karlsson EK, Baranowska I, Wade CM, Salmon Hillbertz NH, Zody MC, Anderson N, et al. Efficient mapping of mendelian traits in dogs through genome-wide association. Nat Genet. 2007;39(11):1321–8. 10.1038/ng.2007.10 17906626

[20] Pedersen NC, Brucker L, Tessier NG, Liu H, Penedo MCT, Hughes S, et al. The effect of genetic bottlenecks and inbreeding on the incidence of two major autoimmune diseases in Standard Poodles, Sebaceous adenitis and Addison’s disease. J Canine Genetics Epidemiol. 2015;2:14. 10.1186/s40575-015-0026-5 PMC4579369 26401342

[21] Amos W, Wilmer JW, Fullard K, Burg TM, Croxall JP, Bloch D, et al. The influence of parental relatedness on reproductive success. Proc R Soc Lond B. 2001;268(1480):2021–7. 10.1098/rspb.2001.1751 PMC1088844 11571049

[22] Acevedo-Whitehouse K, Gulland F, Greig D, Amos W (2003). Disease susceptibility in California sea lions. Inbreeding influences the response of these animals to different pathogens in the wild. Nature.

[23] Hoffman JI, Boyd IL, Amos W (2004). Exploring the relationship between parental relatedness and male reproductive success in the Antarctic fur seal Arctocephalus gazella. Evolution.

[24] Bean K, Amos W, Pomeroy PP, Twiss SD, Coulson TN, Boyd IL (2004). Patterns of parental relatedness and pup survival in the grey seal (Halichoerus grypus). Mol Ecol.

[25] Banks SC, Dubach J, Viggers KL, Lindenmayer DB (2009). Adult survival and microsatellite diversity in possums: effects of major histocompatibility complex-linked microsatellite diversity but not multilocus inbreeding estimators. Oecologia.

[26] Forstmeier W, Schielzeth H, Mueller JC, Ellegren H, Kempenaers B (2012). Heterozygosity–fitness correlations in zebra finches: microsatellite markers can be better than their reputation. Mol Ecol.

[27] Wilbe M, Andersson G (2012). MHC class II is an important genetic risk factor for canine systemic lupus erythematosus (SLE)-related disease: implications for reproductive success. Reprod Domest Anim.

[28] Bloore R (2014). Ulisse. Ital Greyhound Mag.

[29] Trotter PC (2008). Born to Win, Breed to Succeed.

[30] Fretwell F (2014). Colour In the Italian Greyhound. Ital Greyhound Mag.

[31] Hindorff LA, Sethupathy P, Junkins HA, Ramos EM, Mehta JP, Collins FS, et al. Potential etiologic and functional implications of genome-wide association loci for human diseases and traits. Proc Natl Acad Sci U S A. 2009;106(23):9362–7. 10.1073/pnas.0903103106 PMC2687147 19474294

[32] Corona E, Dudley JT, Butte AJ (2010). Extreme evolutionary disparities seen in positive selection across seven complex diseases. PLoS One.

[33] Casto AM, Feldman MW (2011). Genome-wide association study SNPs in the human genome diversity project populations: does selection affect unlinked SNPs with shared trait associations?. PLoS Genet.

[34] Jansson M, Laikre L (2013). Recent breeding history of dog breeds in Sweden: modest rates of inbreeding, extensive loss of genetic diversity and lack of correlation between inbreeding and health. J Anim Breeding Genetics.

[35] Farrell LL, Schoenebeck JJ, Wiener P, Clements DN, Summers KM. The challenges of pedigree dog health: approaches to combating inherited disease. Canine Genetics Epidem. 2015; 2(3):doi:10.1186/s40575-015-0014-9. 10.1186/s40575-015-0014-9 PMC4579364 26401331

[36] Osborne CA, Clifford DH, Jessen C (1967). Hereditary esophageal achalasia in dogs. J Am Ve Med Assoc.

[37] Cox VS, Wallace LJ, Anderson VE, Rushmer RA (1980). Hereditary esophageal dysfunction in the miniature Schnauzer dog. Am J Vet Res.

[38] Irion DN, Schaffer AL, Famula TR, Eggleston ML, Hughes SS, Pedersen NC (2003). Analysis of genetic variation in 28 dog breed populations with 100 microsatellite markers. J Hered.

[39] Haas-Rochholz H, Weiler G (1997). Additional primer sets for an amelogenin gene PCR-based DNA-sex test. Int J Legal Med.

[40] Wictum E, Kun T, Lindquist C, Malvick J, Vankan D, Sacks B (2013). Developmental validation of DogFiler, a novel multiplex for canine DNA profiling in forensic casework. Forensic Sci Int Genet.

[41] Toonen RJ, Hughes S (2002). Increased throughput for fragment analysis on AI prism 377 automated sequencer using a membrane comb and STRand software. Biotechniques.

[42] Stephens M, Smith NJ, Donnelly P (2001). A new statistical method for haplotype reconstruction from population data. Am J Hum Genet.

[43] Boyko AR, Boyko RH, Boyko CM, Parker HG, Castelhano M, Corey L, et al. Complex population structure in African village dogs and its implications for inferring dog domestication history. Proc Natl Acad Sci U S A. 2009;106(33):13903–8. 10.1073/pnas.0902129106 PMC2728993 19666600

[44] Aguilar A, Roemer G, Debenham S, Binns M, Garcelon D, Wayne RK (2004). High MHC diversity maintained by balancing selection in an otherwise genetically monomorphic mammal. Proc Natl Acad Sci U S A.

[45] Wagner JL, Burnett RC, Storb R (1996). Molecular analysis of the DLA DR region. Tissue Antigens.

[46] Morris KM, Kirby K, Beatty JA, Barrs VR, Cattley S, David V, et al. Development of MHC-Linked Microsatellite Markers in the Domestic Cat and Their Use to Evaluate MHC Diversity in Domestic Cats, Cheetahs, and Gir Lions. J Hered. 2014;105(4):493–505. 10.1093/jhered/esu017 PMC4048552 24620003

[47] Peakall R, Smouse PE (2012). Genalex 6: genetic analysis in Excel. Population genetic software for teaching and research. Bioinformatics.

[48] Wright S (1922). Coefficients of inbreeding and relationship. Am Nat.

[49] Queller DC, Goodnight KF (1989). Estimating relatedness using genetic markers. Evolution.

[50] Sacks BN, Brown SK, Stephens D, Pedersen NC, Wu JT, Berry O (2013). Y chromosome analysis of dingoes and southeast asian village dogs suggests a Neolithic continental expansion from Southeast Asia followed by multiple Austronesian dispersals. Mol Biol Evol.

[51] Lacy RC, Analysis of founder representation in pedigrees (1989). Founder equivalents and founder genome equivalents. Zoo Biol.

